# Controlling the strontium-doping in calcium phosphate microcapsules through yeast-regulated biomimetic mineralization

**DOI:** 10.1093/rb/rbw025

**Published:** 2016-07-31

**Authors:** Miaojun Huang, Tianjie Li, Ting Pan, Naru Zhao, Yongchang Yao, Zhichen Zhai, Jiaan Zhou, Chang Du, Yingjun Wang

**Affiliations:** ^1^School of Materials Science and Engineering, South China University of Technology, Guangzhou 510641, China;; ^2^National Engineering Research Center for Tissue Restoration and Reconstruction, Guangzhou 510006, China; ^3^Ministry of Education Key Laboratory of Biomedical Materials Science and Engineering, Guangzhou 510006, China

**Keywords:** calcium phosphate, yeast, biotemplate, strontium

## Abstract

Yeast cells have controllable biosorption on metallic ions during metabolism. However, few studies were dedicated to using yeast-regulated biomimetic mineralization process to control the strontium-doped positions in calcium phosphate microcapsules. In this study, the yeast cells were allowed to pre-adsorb strontium ions metabolically and then served as sacrificing template for the precipitation and calcination of mineral shell. The pre-adsorption enabled the microorganism to enrich of strontium ions into the inner part of the microcapsules, which ensured a slow-release profile of the trace element from the microcapsule. The co-culture with human marrow stromal cells showed that gene expressions of alkaline phosphatase and Collagen-I were promoted. The promotion of osteogenic differentiation was further confirmed in the 3D culture of cell-material complexes. The strategy using living microorganism as ‘smart doping apparatus’ to control incorporation of trace element into calcium phosphate paved a pathway to new functional materials for hard tissue regeneration.

## Introduction

There is an increasing scientific interest in the development of synthetic systems for functional inorganic materials to tailor their composition, morphology and performance for various engineering requirements [1–4]. From morphology perspective, many non-toxic inorganic materials had been formed into nano or micro-scaled sphere shape with hollow structure to have larger surface area and capacity to load or encapsulate more therapeutic agents such as metallic ions, and then applied in medical field. Templating method have attracted much attention in the fabrication of so-called capsule-like structure. Many efforts had been took to find an effective and non-toxic template, such as CTAB [5] and polymeric micelle [6], to form capsule-like structure in calcium phosphate biomedical materials to make them more applicable in drug delivery system. Doping of minor elements to control the composition can further enhance the functionality of the materials [7–9]. For calcium phosphate biomedical materials, doping or incorporation of ions (such as Zn^2+^, Mg^2+^, Sr^2+^, F^−^, ${\text{CO}}_{\text{3}}^{\text{2}}$^−^, etc.) can significantly reinforce their biological properties due to a closer similarity with the natural bone mineral [10–14].

Strontium is stored in skeleton by exchanging with calcium ions in the hydroxyapatite crystal lattice, preferably in new trabecular bone and with variations depending upon the skeletal site [15]. It has been confirmed that low dose of strontium has effect on both reducing bone resorption and increasing uptake of calcium into bones or stimulating bone formation [16]. Nevertheless, high dose of strontium have poisonous effects on bone mineralization due to its occupation of the sites where calcium would be, leading to the reduction of calcium adsorption of bones [17]. So continuous release at an appropriate low level of strontium from bone repair materials could have promoting effect on osteogenesis. Various synthetic methods have been studied to incorporate strontium into calcium phosphate, such as solid state calcination [18], co-precipitation [19], sol–gel process [20] and hydrothermal method [21]. For solid phase method, a subsequent milling process is necessary to completely mix the raw materials. Most traditional liquid phase method can only prepare the sheet/rod-like powders and the effective control over the composition, structure and morphology is still a challenge. Furthermore, it remains demanding to control the distribution and release of strontium to maximize its biological function.

The living microorganism is attracting attention for preparing functional materials through biomimetic processes [22–24]. Yeast cells (YCs) have been employed as biotemplate to fabricate porous inorganic microsphere or microcapsules [25–27]. In our recent work, strontium had also been successfully doped into porous calcium phosphate microcapsules (CPMCs) by combining YCs as biotemplate and solution co-precipitation [28]. However, during the solution co-precipitation process, the amount of Sr doped in the calcium phosphate mineral increased linearly with the original Sr ion concentration in the solution and YCs would have little control on the doping process except serving as a passive template [28]. In fact, YCs are remarkable for their special biosorption towards various heavy metal ions, such as Cu^2+^, Pb^2+ ^and Ni^2+^ [29, 30], although the biosorption is not efficacious for all metallic ions [30]. There is also a few reports about the absorption effect of YCs on Sr^2+^ [31]. Considering the significant biological function of Sr^2+ ^and the limitation of traditional methods in composition or morphological control, we further our study to use yeast-regulated biomimetic mineralization process to synthesize strontium-doped CPMCs.

In this work, we introduced YCs to pre-adsorb strontium ions metabolically and then served as the sacrificing template for the precipitation and calcination of mineral shell. YCs served as both mineralization biotemplate and the ion-doping apparatus. The control on loading dose and enriching position of Sr^2+ ^in microcapsule were investigated. The biological performance of the product was evaluated by using human marrow stromal cells (hMSCs). The biosorption method and final product are potentially applicable for the development of advanced functional inorganic materials.

## Experimental section

### Synthesis of CPMC

**Figure 1.**
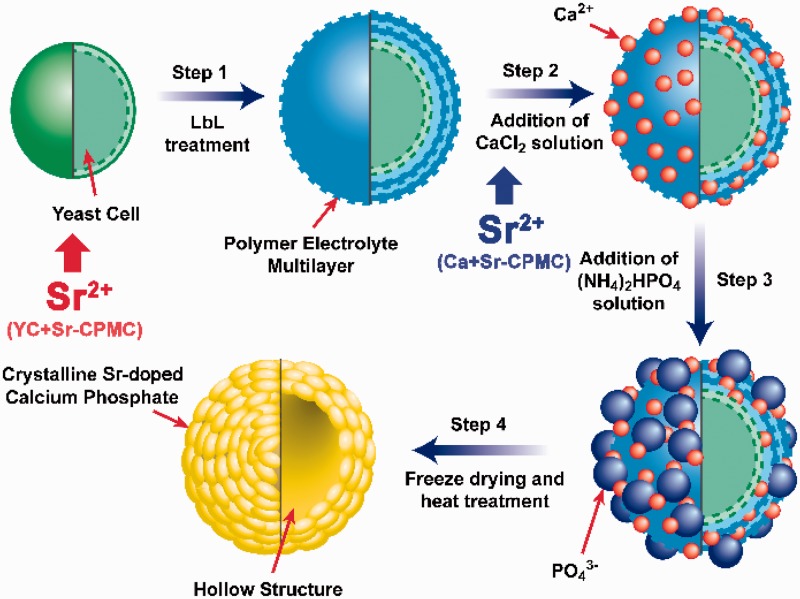
Procedure involved in the preparation of YC + Sr-CPMC and Ca + Sr-CPMC

Baker’s yeast (*Saccharomyces cerevisiae*) cells were isolated and purified from the ANGEL’s instant dry yeast by the spread plate method and the streak plate method. YCs were cultured in yeast extract peptone dextrose medium with SrCl_2_ solutions of different original concentrations (Fig. 1 red), incubated at 28°C with continuous shaking at 180 rpm. The appropriate cultured YCs were alternately immersed into 1 wt% Poly(diallyl-dimethylammonium chloride) (PDADMAC) solution and 1 wt% Poly(acrylic acid) (PAA) solution via the layer-by-layer (LbL) treatment and formed a yeast/PDADMAC/PAA composite biotemplate. The biotemplates were mixed with CaCl_2_ solution and then (NH_4_)_2_HPO_4_ solution after centrifugation, stirring for 12 h under ambient temperature, respectively. The pH value was regulated to ∼10. After centrifugation and washing, the product was dried and then calcined at 800°C. The corresponding products with different original SrCl_2_ concentrations (0, 0.4, 1.0, 2.0 and 10.0 mg/ml) were denoted as YC + Sr0, YC + Sr0.4, YC + Sr1.0, YC + Sr2.0 and YC + Sr10.0, respectively.

For comparison, the YCs were firstly cultured without Sr-addition, followed by LbL treatment and mineralization using the co-precipitation process as reported in our previous work [28]. The CaCl_2_ solution was replaced by the CaCl_2_ and SrCl_2_ mixed solution (Fig. 1 blue). This process was similar to the common chemical co-precipitation synthesis of Sr-doped calcium phosphate powders [32, 33]. The powders were autoclaved and stored in a sealed tube. Two different molar ratios of Sr/(Sr + Ca) in the mixed solution of CaCl_2_ and SrCl_2_ were used as 1 and 5 at%. The corresponding products were denoted as YC + Sr0/Ca + Sr1 and YC + Sr0/Ca + Sr5, respectively.

The following four samples were tested to further study the effect of YCs regulation on Sr^2+ ^enrichment location in Sr-doped CPMCs: YC + Sr0 (control sample of pure CPMC without strontium), YC + Sr2.0, YC + Sr0/Ca + Sr5 and YC + Sr2.0/Ca + Sr5. The sample YC + Sr2.0/Ca + Sr5 is a combination of two Sr^2+ ^doping methods, i.e. YCs were firstly cultured with addition of SrCl_2_ solutions (2.0 mg/ml), followed by the mineralization in a mixed solution of CaCl_2_ and SrCl_2_ (Sr/(Sr + Ca) = 5 at% molar ratio). In addtion, the following two samples were tested to study the elemental composition of the whole particles: YC + Sr1.0 and YC + Sr0/Ca + Sr1, which were used for the subsequent cell experiments.

### Material characterization

X-ray diffraction (XRD) patterns of samples were obtained on PANalytical X’ Pert PRO (PANalytical, Netherlands) by using Cu Kα radiation (λ = 0.15418 nm). The morphologies of the products were observed by the scanning electron microscopy (SEM, Hitachi H-800). The qualitative and semi-quantitative element analysis of the particle sample was tested by using the energy dispersive spectroscopy (EDS, EPMA-1600, Shimadzu Corporation) and X-ray photoelectron spectroscopy (XPS, Axis Ultra, Shimadzu Corporation). The elemental composition of the whole particle was tested by using the atomic absorption spectrometer (AAS, PE-AA400, PerkinElmer Corporation) after dissolving in HNO_3_.

### Sr-release behaviour measurement

In total, 15 mg CPMCs, 15 mg YC + Sr-CPMCs and 15 mg Ca + Sr-CPMCs were added into separated 15 ml Dulbecco’s Modified Eagle’s Medium with low glucose (L-DMEM), respectively, continuous shaking at 37°C. Sr-release behaviour of Sr-CPMCs in L-DMEM was investigated by measuring the strontium ion concentrations of the solutions at different time points. At each time point, 5 ml supernatant was taken from each sample for measurement. After sampling, the samples were filled to 15 ml with L-DMEM. The strontium ion concentration was determined by means of the inductive coupled plasma emission spectrometer (ICP, PS1000-AT, Perkin Elmer Corporation). For statistics, three samples were assayed at each time point (1, 3, 7, 14 day), respectively.

### Cell experiment

The hMSCs were donated from the First Affiliated Hospital of Sun Yat-sen University in Guangzhou as described in the reference [34]. Cells were exposed to various CPMC samples via the following way. The powders were suspended with the low sugar complete medium under ultrasonic treatment to a predetermined final concentration (200 μg/ml). The cell suspension of hMSCs was added into the CPMC suspension to a specified concentration range (4 × 10^3^ cells/hole in 96-well plate). The mixture was feed and placed in incubator (37°C, 5% CO_2_). The Cell Counting Kit-8 (CCK-8) was used to conduct the cell proliferation and cytotoxicity experiment. The cell suspension without powder and the one with 0.65% phenol solution were referred as tissue culture plate control and phenol control, respectively. All samples were assayed at certain time points (4 hour, 1 day, 3 day and 7 day).

For the cellular differentiation assay, cells were exposed to various CPMC powders (2 × 10^5^ cells/hole in 6-well plate) and cultured for 3 days. The osteogenic medium was used to induce hMSCs differentiation *in vitro*. The samples were tested by the alkaline phosphatase (ALP) assay and the gene expression assay.

The CCK-8 and ALP activity assays were tested by using the multifunctional microplate reader (Thermo3001, U.S. Thermo Electron Corporation). ALP staining was conducted by using the BCIP/NBT phosphatase substrate (1-Component) (KPL, USA). The stained samples were observed by using a digital 3D video microscope (HIROX KH-7700, Japan). The gene expression assay was operated by using the fluorescence quantitative polymerase chain reaction (PCR) instrument (Chromo 4, American Bio-Rad Company).

### 3D co-culture of Cell-CPMC complex

Equal amount of powders of YC + Sr0, YC + Sr1.0 and YC + Sr0/Ca + Sr1 were added to cell suspensions of hMSCs, respectively. The mixtures were co-cultured under osteogenic induction in EP tubes for 30 days, to form 3D Cell-CPMC complex. SEM was used to observe the morphology of the complex. The cell-material complex was fixed and stained by Coomassie brilliant blue staining for observation under the light microscope. Immunohistochemical analysis of Collagen-I production was conducted on the tissue sections with Collagen-I staining kit to further study the biological performances of Cell-CPMC complexes.

## Results and discussion

The biosorption of Sr^2+ ^ions on YCs was studied by culturing yeasts in SrCl_2_ solutions of different concentrations for 4 days (Supplementary Fig. S1). The uptake of Sr^2+ ^was relatively fast in the first 2 days, and then leveled off at the third day when the adsorption of Sr^2+ ^was saturated. With the increase of initial SrCl_2_ concentration, the saturated amount of Sr^2+ ^absorbed by YCs increased accordingly. The results suggested that YCs can adapt themselves to the environment and adjust their biosorption ability for metal ions. Sr^2+ ^was involved in both ionic and covalent bonding to the oxygen-containing ligands on the YC wall [35]. For the subsequent mineralization, the YCs being cultured for 3 days were used as biotemplate.

**Figure 2.**
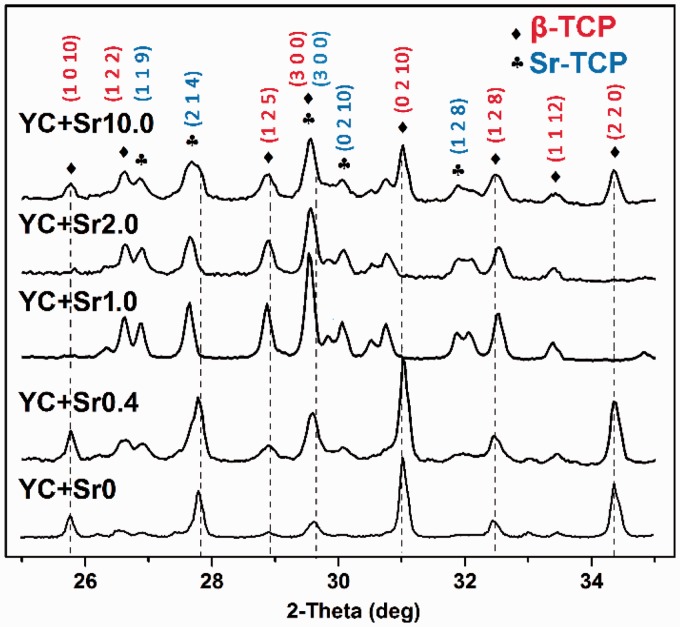
XRD patterns of calcium phosphate products after YC-regulated mineralization and calcinations. The YCs were allowed to pre-adsorb Sr^2+ ^ions in SrCl_2_ solutions of different concentrations. The corresponding products with different original SrCl_2_ concentrations (0, 0.4, 1.0, 2.0 and 10.0 mg/ml) were denoted as YC + Sr0, YC + Sr0.4, YC + Sr1.0, YC + Sr2.0 and YC + Sr10.0, respectively

The YCs pre-adsorbing Sr^2+ ^ions proved to be effective doping apparatus for the incorporation of Sr^2+ ^into calcium phosphate materials. As shown in the XRD patterns (Fig. 2), without Sr^2+ ^ions (YC + Sr0), YC-templated mineralization and calcination led to the formation of Whitlockite-β-tricalcium phosphate (β-TCP, JCPDS card No. 00-009-0169). After adsorption of Sr^2+ ^ions, the sample YC + Sr0.4 presented peaks of Sr-TCP (JCPDS card No. 00-052-0467) though β-TCP was still the dominant phase. However, once the initial Sr^2+ ^concentration increased to 1.0 and 2.0 mg/ml, Sr-TCP became the dominant phase and the characteristic peaks of β-TCP offset. Some of characteristic peaks of β-TCP got strengthened, such as (300) and (125) in YC + Sr1.0 and YC + Sr2.0. This result was expected considering the differences between both cationic radius and incorporation position. This phenomenon manifests that Sr^2+ ^(1.25 Å for 8-fold coordination with O), with larger cationic radius than Ca^2+ ^(1.12 Å for 8-fold coordination with O), incorporated into calcium phosphate crystalline lattice and substituted Ca^2+^, giving rise to an increase in lattice distance and distortion of lattice. In β-TCP lattice structure, there are five different Ca sites. From Rietveld refinement studies [36, 37], Sr^2+ ^preferentially occupies the 9-fold coordinated Ca (4) sites of β-TCP structure, which has a partial occupancy factor of 0.5. This is due to the longest Ca (4)-O (9) mean distance (3.041 and 3.228 Å) in the β-TCP structure could easily accommodate large sized Sr^2+ ^at Ca (4) sites.

Table 1 presents the lattice paramenters a and c of the Sr-TCP structure. The incorporation of Sr^2+ ^into TCP crystalline lattice can increase the unit parameter a and c [32], which were increased along with the initial concentration of SrCl_2_ in YPD medium. The substitution of Ca atom with larger Sr atom stablizes the structure of β-TCP [20], leading to β-TCP crystals growing along certain orientation.

**Table 1.** Lattice parameters of Sr-TCP structure

Sample	a (Å)	c (Å)
YC+Sr0	10.42618	37.37138
YC+Sr0.4	10.43200	37.38552
YC+Sr1.0	10.43230	37.47840
YC+Sr2.0	10.43240	37.51781
YC+Sr10.0	10.43503	37.37682

It is interesting to note that both β-TCP and Sr-TCP phases dominated in the case of YC + Sr10.0, despite that YCs had the highest initial biosorption on Sr^2+ ^in this case (Supplementary Fig. S1). Further characterization of the content of strontium in the calcium phosphate products by EDS analysis (Supplementary Fig. S2) was in good consistency with the results of XRD. The EDS patterns of the samples prepared with different initial SrCl_2_ concentrations show that the content of strontium in the calcium phosphate products first increased and then decreased with the increasing initial SrCl_2_ concentrations, and the peak presented at around 2.0 mg/ml (Supplementary Fig. S2). Although YCs can absorb more strontium when cultured in SrCl_2_ solution of higher concentration, the increased loading of strontium may have detrimental effects on the cells, leading to their denaturation and even death. It has been suggested that the denaturation process would inhibit the functional groups on the cell wall, which were involved in covalent bonding of Sr^2+^, to prevent further action in dead cells [35]. The overall bonding strength of Sr^2+ ^to the cells would be weakened and Sr^2+ ^would then easily lost from the cells during the following LbL treatment and mineralization process. This could explain a lower level of Sr doping detected in YC + Sr10.0 samples.

**Figure 3.**
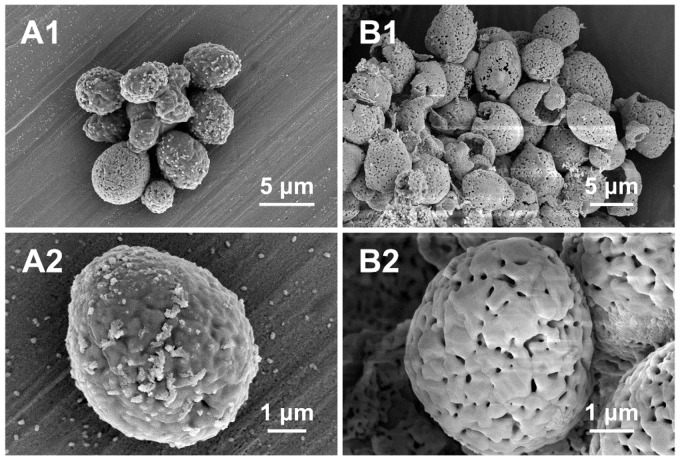
Typical SEM images of YC + Sr1.0 CPMC before **(A)** and after **(B)** calcination at 800 °C

The typical SEM images of YC + Sr1.0 show that the synthesized microcapsules with diameter of ∼5 μm almost maintained the size and spherical morphology after calcination at 800°C (Fig. 3). The final products have a hollow structure due to the removal of yeast biotemplate as illustrated from the broken microcapsules (Fig. 3B1). Nano-pores with diameter of ∼100 nm can be observed on the surface of the calcined microcapsules (Fig. 3B2).

**Figure 4.**
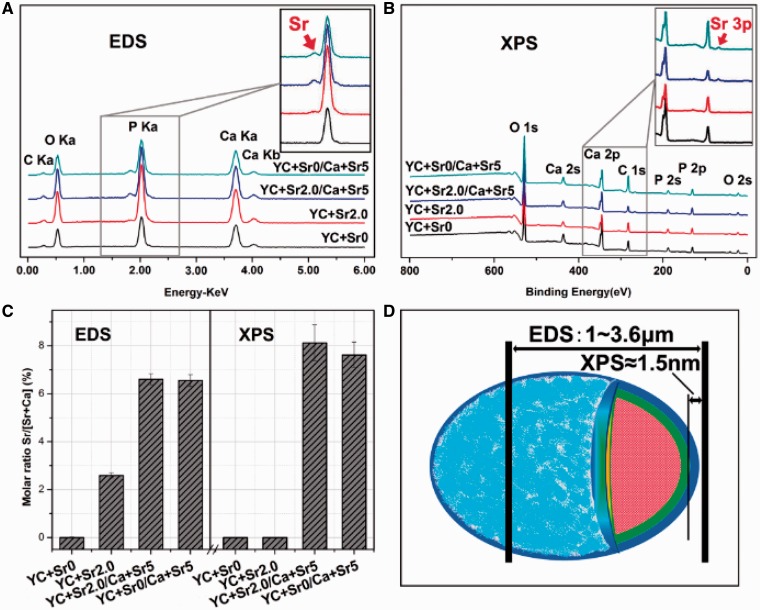
EDS and XPS patterns of various CPMCs with different synthesis processes: **(A)** EDS patterns; **(B)** XPS patterns; **(C)** molar ratio of Sr/(Sr+Ca); **(D)** schematic diagram showing the detecting depth of EDS and XPS

EDS and XPS analysis were combined to investigate the enrichment of strontium within the microcapsule (Fig. 4). For comparison, YC + Sr0, YC + Sr2.0, YC + Sr0/Ca + Sr5 and YC + Sr2.0/Ca + Sr5 were tested to reveal the control effect of YCs. The results of EDS (Fig. 4A and C) show that strontium were detected in all samples except YC + Sr0. Sr^2+ ^content of YC + Sr0, YC + Sr2.0, YC + Sr0/Ca + Sr5 and YC + Sr2.0/Ca + Sr5, expressed as the molar ratio of Sr/(Sr + Ca), were 0 ± 0.02 at%, 2.59 ± 0.11 at%, 6.60 ± 0.24 at% and 6.56 ± 0.24 at%, respectively. In contrast, XPS analysis (Fig. 4B and C) detected strontium in YC + Sr0/Ca + Sr5 and YC + Sr2.0/Ca + Sr5 but not YC + Sr0 and YC + Sr2.0. Sr^2+ ^content of YC + Sr2.0/Ca + Sr5 and YC + Sr0/Ca + Sr5 was 8.12 ± 0.76 at% and 7.63 ± 0.53 at%, respectively, which was significantly higher than Sr^2+ ^concentrations detected by EDS.

The detecting depth of EDS is in the micron scale, while that of XPS is within nanometer scale (Fig. 4D). The combined results of EDS and XPS for YC + Sr2.0 sample thus suggested that Sr^2+ ^was mainly enriched in the inner layer of biomimetic synthetic microcapsules. The co-precipitation process for YC + Sr0/Ca + Sr5 through polyelectrolyte adsorption led to an enrichment of Sr^2+ ^mainly in the outer layer of microcapsules.

Table 2 presents the elemental composition of the whole particle by AAS test after dissolving in nitric acid. It shows that there were similar weight percentage for Sr between the two samples, the subsequent comparison studies were conducted with YC + Sr1.0 and YC + Sr0/Ca + Sr1.

**Table 2.** The elemental composition (Ca, Sr and P)

Sample	Ca (wt%)	Sr (wt%)	P (wt%)
YC+Sr1.0	35.04 ± 4.15	0.90 ± 0.14	18.27 ± 2.17
YC+Sr0/Ca+Sr1	34.01 ± 2.97	1.06 ± 0.14	17.77 ± 1.56

**Figure 5.**
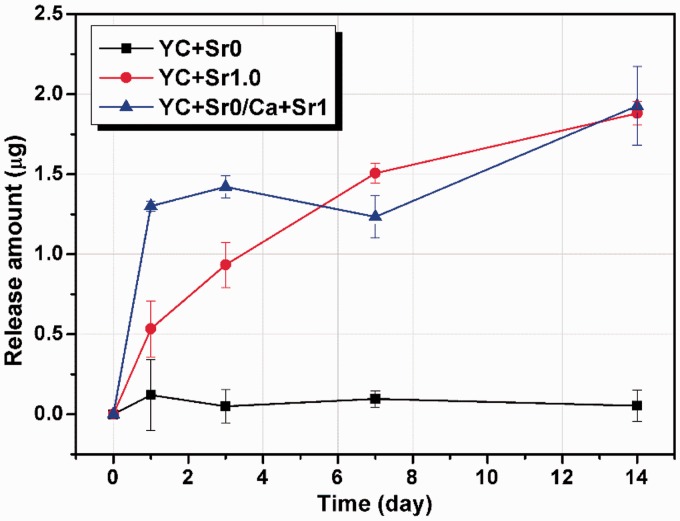
Sr^2+ ^release amount curves of YC + Sr0, YC + Sr1.0 and YC + Sr0/Ca + Sr1 in L-DMEM

The release behaviours of Sr^2+ ^in L-DMEM from YC + Sr0, YC + Sr1.0 and YC + Sr0/Ca + Sr1 were further investigated (Fig. 5). For YC + Sr0/Ca + Sr1 in which strontium was enriched in the surface of the microcapsules, there was an apparent burst release at Day 1 followed by a plateau. In comparison, YC + Sr1.0 presented a relatively steady slow-release pattern. The different enrichment position has certain effect on the release pattern.

**Figure 6.**
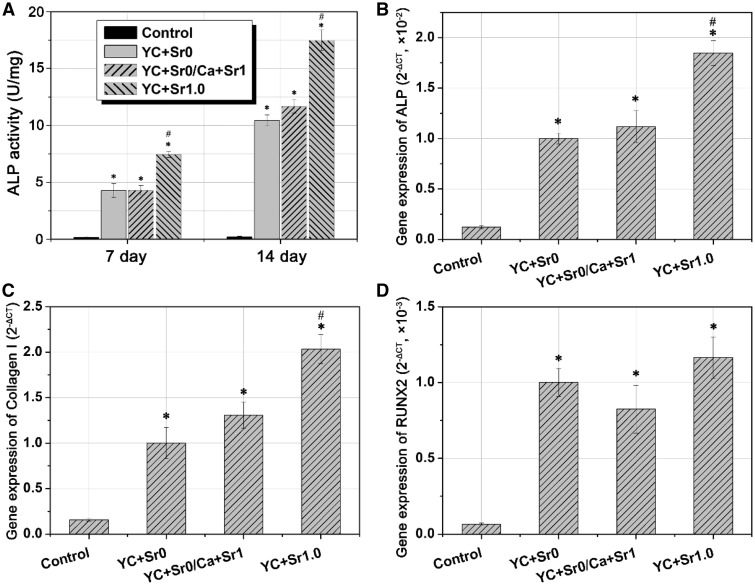
**(A)** ALP activity of hMSCs on different CPMCs at 7 and 14 days and RT-PCR analysis of **(B)** ALP, **(C)** Collagen-I and **(D)** RUNX2 genes from different CPMCs groups at Day 7. (* and # indicate statistical significance compared with control and YC + Sr0, respectively. *P* < 0.05). the control sample refers to YC + Sr0 without osteogenic induction medium

For YC + Sr1.0 and YC + Sr0/Ca + Sr1 both benefited the proliferation of hMSCs and possessed little cytotoxicity (Supplementary Fig. S3), their ability to induce osteogenetic differentiation of hMSCs were further studied. ALP activity per cell at Days 7 and 14 during incubation was measured by pNPP method to investigate the degree of differentiation of hMSCs into osteoblastic cells (Fig. 6A). The strontium-containing groups of YC + Sr0/Ca + Sr1 showed slightly higher ALP activity than the pure CPMC (YC + Sr0) at Day 14, while YC + Sr1.0 stimulated even higher ALP activity at Days 7 and 14. The result of ALP activity test conformed well to that of the ALP staining test (Supplementary Fig. S4). In general, ALP test indicated that strontium-containing calcium phosphate has the ability to stimulate the osteogenic differentiation of hMSCs and the incorporation of Sr^2+ ^into CPMC through yeast biosorption seems to be more effective.

Reverse transcription (RT)-PCR was used to further analyse the gene expression (ALP, Collagen-I and RUNX2) of the osteogenic differentiation (Fig. 6B–D). It can be clearly seen that the majority of osteogenesis-related genes expression was up regulated by strontium-containing CPMC, and YC + Sr1.0 showed significantly enhanced effect than YC + Sr0/Ca + Sr1.

**Figure 7.**
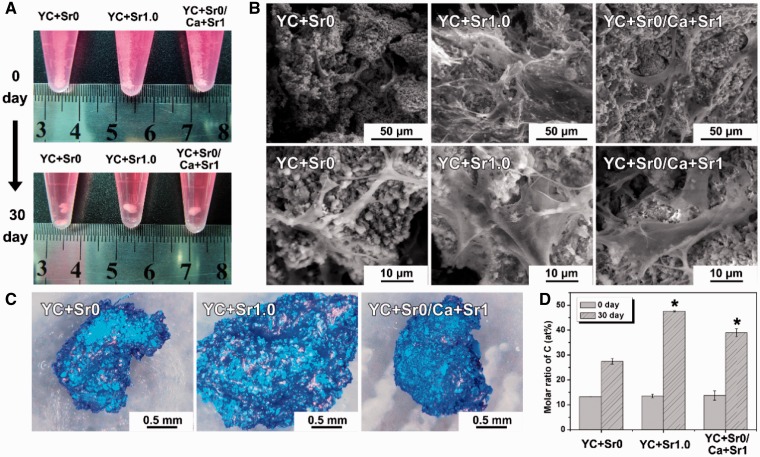
**(A)** The photographs of hMSCs cultured with various CPMCs at 0 and 30 days; **(B)** the SEM morphologies of Cell-CPMC complexes at Day 30; **(C)** the 3D morphologies of Cell-CPMC complexes after coomassie brilliant blue staining at Day 30; **(D)** the EDS patterns of the molar ratio of C element in Cell-CPMC complexes at 0 and 30 days. (* indicates statistical significance compared with YC + Sr0. *P* < 0.05)

For 3D co-culture of Cell-CPMC complex, white powder of all CPMC groups dispersedly precipitated in EP tubes before hMSCs inoculation. After hMSCs were seeded and co-cultured with the materials for 30 days, the CPMC powder and cells bound together, forming a 3D complex (Fig. 7A). The complex can suspend in the culture medium with a size of around 3 mm in diameter. The Cell-CPMC complex was cut for SEM observation to further observe the internal structure (Fig. 7B). Cells can be observed at both surface and internal of the complexes. The cells spread out on the materials especially on YC + Sr1.0 and YC + Sr0/Ca + Sr1. The results suggest that all kinds of CPMC powder have good cytocompatibility and binds tightly to hMSCs. With this co-culture model, cells are fully integrated with the powder into a composite unity, providing a 3D space suitable for their own growth and proliferation. The cells can adhere and proliferate on the CPMC granules, stick to and bind together the powder aggregates to form a 3D composite cluster after prolonged incubation.

Cell-CPMC complexes were fixed, fully dehydrated, prepared by Coomassie brilliant blue staining and observed by the 3D microscope (Fig. 7C). All samples maintained their 3D structure with a slight shrinking. And all complexes presented bright blue, in which dark blue indicated organics and light blue for CPMC materials. It suggested a large amount of protein existed in complexes. The EDS results further showed that there was more carbon element in YC + Sr1.0 at 30 day than YC + Sr0 and YC + Sr0/Ca + Sr1 samples (Fig. 7D), which implied that more organic matter were formed in the case of YC + Sr1.0.

**Figure 8.**
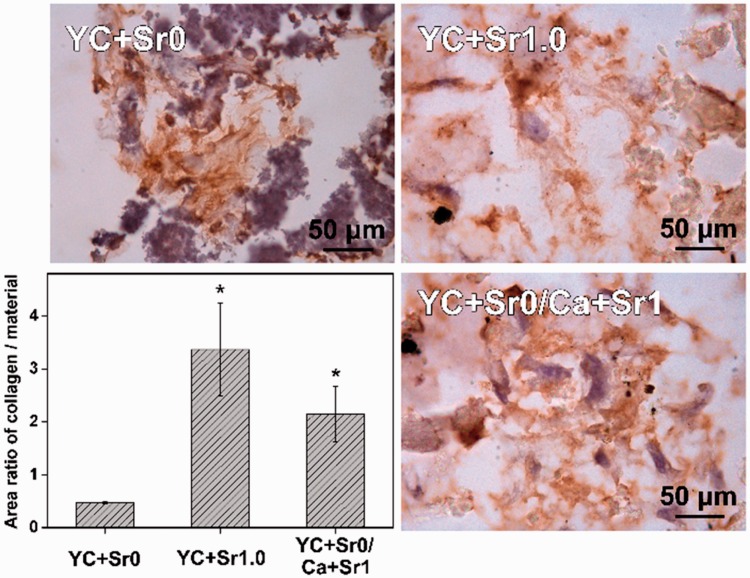
Representative Collagen-I immunohistochemical staining analysis of Cell-CPMC complexes. The bar chart showed the comparison of average area ratio of collagen/material from randomly selected photographs (*n* = 5). (* indicates statistical significance compared with YC + Sr0. *P* < 0.05)

Collagen-I immunohistochemical staining was utilized on the tissue sections to further investigate the effect of the Sr-CPMC on the production of bone matrix. For a quantitative evaluation, the area ratio of collagen to materials was calculated from randomly selected sections (Fig. 8). The area ratios of collagen to materials of YC + Sr1.0 and YC + Sr0/Ca + Sr1 are significantly larger than that of YC + Sr0. Furthermore, the area ratio of collagen to materials of YC + Sr1.0 samples was even higher than that of YC + Sr0/Ca + Sr1 samples. The results confirmed the well biocompatibility of Sr-CPMC and suggested that YC + Sr1.0 had enhanced effect on the production of collagen matrix than YC + Sr0/Ca + Sr1.

Collectively, Sr-CPMC can closely integrate with hMSCs to form 3D composite complex. The hMSCs showed well adhesion, proliferation and differentiation both inside and outside of the complex during 30 days of culture and osteogenic induction.

## Conclusions

The biosorption of strontium by YCs enables a novel approach for the biomimetic syntheses of porous Sr-doped CPMCs. YCs can modify the inorganic microcapsule with enriching low-dose trace element from inside as well as serving as the sacrificial template for the formation of a hollow structure. The content of Sr^2+ ^can be effectively controlled through the normal metabolism of host cells. Moreover, the doping of trace element from inside the microcapsule led to a relatively steady release pattern instead of a burst release of the doping ions.

Sr-doped CPMC showed excellent cytocompatibility and biological performance of promoting osteogenic differentiation of hMSCs. Interestingly, the Sr-doped CPMC fabricated through yeast-regulated process promoted gene expressions of ALP and Collagen-I, which were significantly higher than that of pure CPMC and the solution precipitated Sr-doped CPMC. In addition, a 3D cell-CPMC complex formed after co-culture of the CPMC powder and hMSCs, with the potential to develop into bone-like tissues. The current strategy using living microorganism as ‘smart doping apparatus’ to control incorporation of the trace element into calcium phosphate is promising for the development of new functional materials for biomedical applications such as bone tissue engineering.

## Supplementary Material

Supplementary Fig. S1
