# Supplementary material for: Controlling the strontium-doping in calcium phosphate microcapsules through yeast-regulated biomimetic mineralization
Source: Regen Biomater. 2016 Jul 31;3(5):269–76. doi: 10.1093/rb/rbw025 (PMC5043151; doi:10.1093/rb/rbw025)
Supplement: Supplementary Fig. S1 [file Supporting_information.docx]

**Supporting Information for:**

**Controlling the Strontium-Doping in Calcium Phosphate Microcapsules through Yeast-Regulated Biomimetic Mineralization**

Miaojun Huang^12,†^, Tianjie Li^13,†^, Ting Pan^13^, Naru Zhao^12^, Yongchang Yao^1^, Zhichen Zhai^1^, Jiaan Zhou^2^, Chang Du^13,^* and Yingjun Wang^12,^*

^1^ School of Materials Science and Engineering, South China University of Technology, Guangzhou 510641, China; ^2^ National Engineering Research Center for Tissue Restoration and Reconstruction, Guangzhou, 510006, China; ^3^ Ministry of Education Key Laboratory of Biomedical Materials Science and Engineering, Guangzhou, 510006, China.

* Correspondence address. School of Materials Science and Engineering, South China University of Technology, Guangzhou 510641, China. Fax: +86-20-22236088; Tel: +86-20-87112160; Email: [duchang@scut.edu.cn](mailto:duchang@scut.edu.cn)

^†^ These authors contributed equally to this work.

**
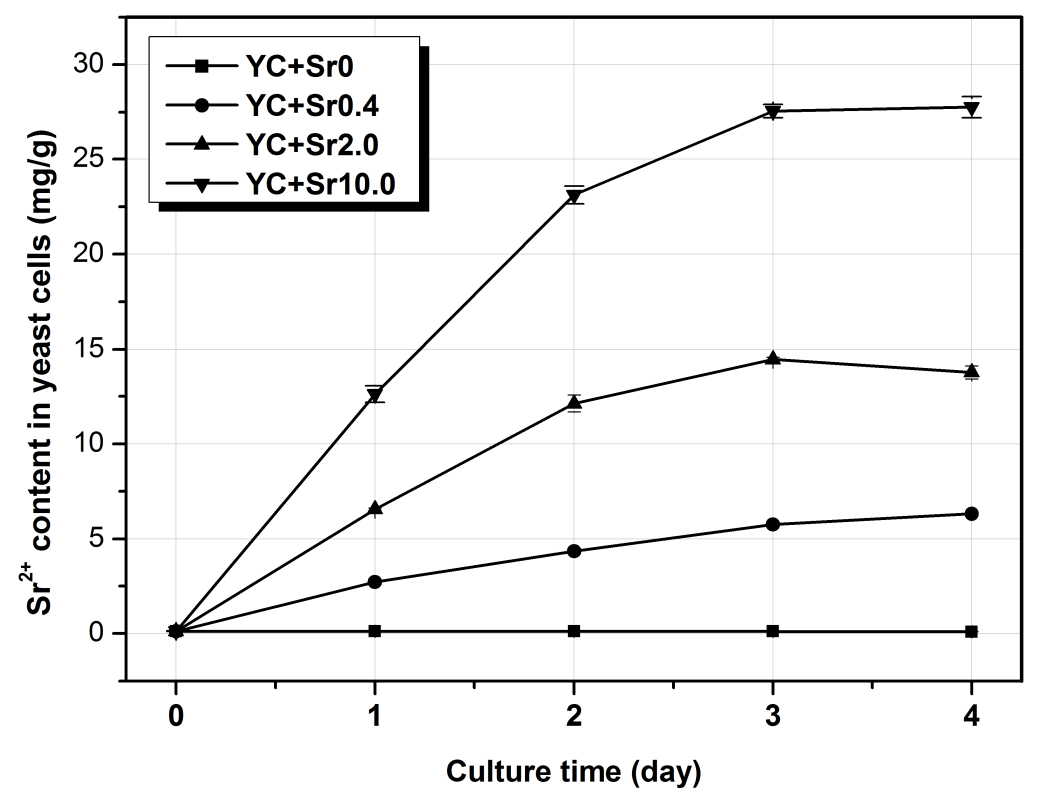
**

**Figure S1. Sr^2+^ content of YCs cultured with different initial SrCl_2_ concentrations. YC+Sr0, YC+Sr0.4, YC+Sr2.0 and YC+Sr10.0 indicated that the concentrations of SrCl_2_ were 0 mg/mL, 0.4 mg/mL, 2.0 mg/mL and 10.0 mg/mL, respectively.**

The Sr^2+^ contents of samples were obtained with atomic absorption spectroscopy (**AAS**, PE-AA400, PerkinElmer). Biosorption of Sr^2+^ by YCs cultured with different initial SrCl_2_ concentrations was studied. The uptake of Sr^2+^ was relatively fast in the first two days, and then leveled off at the third day when the adsorption of Sr^2+^ was saturated. With the increase of initial SrCl_2_ concentration, the saturated amount of Sr^2+^ absorbed by YCs increased accordingly. The results suggested that YCs can adapt themselves to the environment and adjust their biosorption ability for metal ions. For the subsequent mineralization, the YCs being cultured for 3 days were used as biotemplate.

**
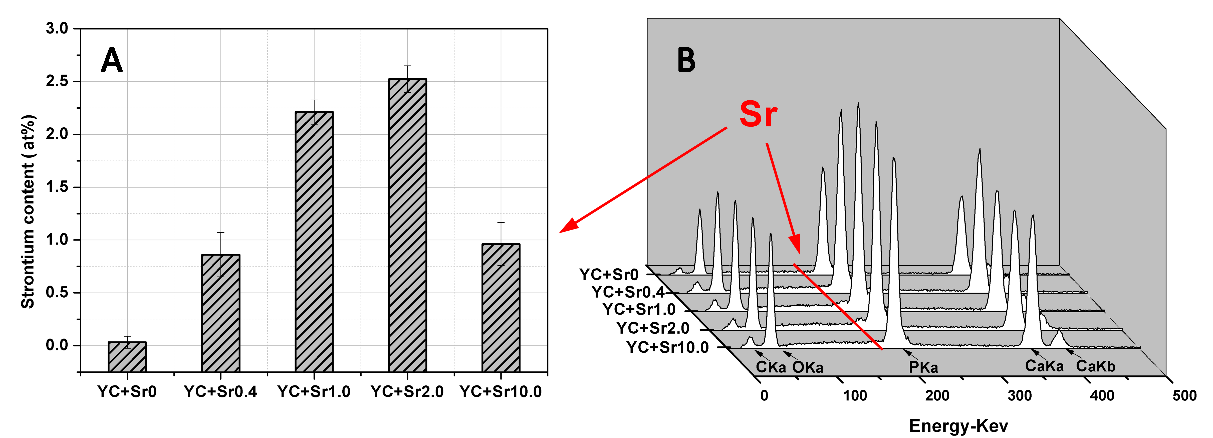
**

**Figure S2. A) The atomic percentage of Sr to Sr + Ca in YC+Sr-CPMC calculated from EDS spectra shown in B. B) The EDS patterns of YC+Sr-CPMC with different initial SrCl_2_ concentrations. The red line showed the characteristic peaks of Sr. YC+Sr0, YC+Sr0.4, YC+Sr1.0, YC+Sr2.0 and YC+Sr10.0 represented for YC+Sr-CPMC with initial SrCl_2_ concentrations of 0 mg/mL, 0.4 mg/mL, 1.0 mg/mL, 2.0 mg/mL and 10.0 mg/mL, respectively.**

The EDS patterns of the samples prepared with different initial SrCl_2_ concentrations show a net presence of strontium incorporated. The results show that the content of strontium in YC+Sr-CPMC first increased and then decreased with the increasing initial SrCl_2_ concentrations, and the peak presented at 1.0-2.0 mg/mL, which is in good consistency with the results of XRD. Although YCs can absorb more strontium when cultured in SrCl_2_ solution of higher concentration, the increased loading of strontium may have detrimental effects on the cells, leading to their denaturation and even death. It has been suggested that the denaturation process would inhibit the functional groups on the cell wall, which were involved in covalent bonding of Sr^2+^, to prevent further action in dead cells (reference 32 in the main text). The overall bonding strength of Sr^2+^ to the cells would be weakened and Sr^2+^ would then easily loosed from the cells during the following LbL treatment and mineralization process. This could explain a lower level of Sr doping detected in YC+Sr10.0 samples. The most pronounced dominance of biomimetic synthesis of Sr-CPMCs through yeast metallic biosorption is that YCs can intelligently absorb strontium and control the amount of metallic ions in CPMCs, which will do little harm to the growth and metabolism of host cells.

**
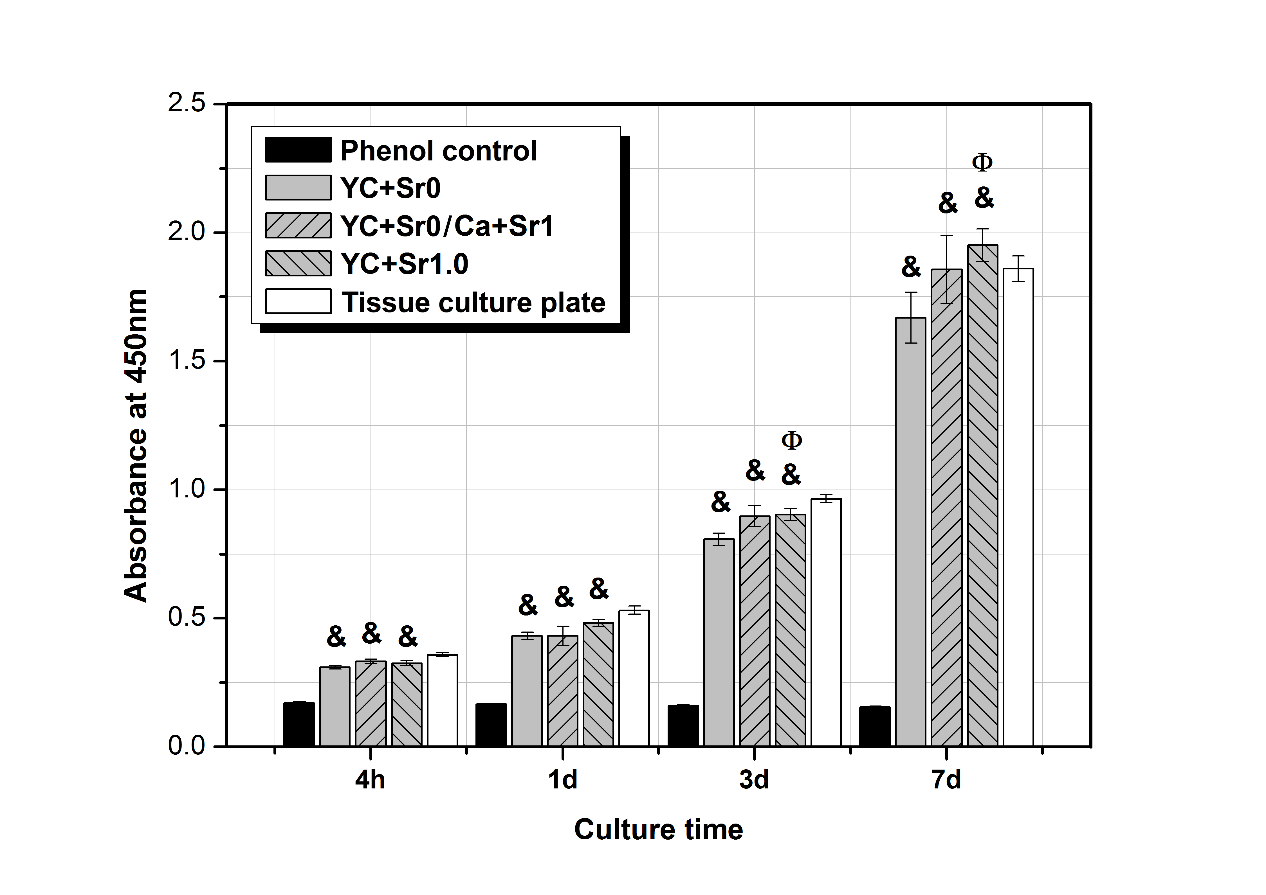
**

**Figure S3. The CCK-8 analysis of CPMCs culturing with hMSCs at 4 h, 1 d, 3 d and 7 d. (& and Ф indicated statistical significance when compared with Phenol control and YC+Sr0, respectively. *P* < 0.05)**

From results of CCK-8 testing, it can be observed that with the increase of incubation time, OD_450_ value of the experimental groups and tissue culture plate increased. The OD_450_ at 7th day of YC+Sr1.0 group existed statistically significant higher value (*P* < 0.05) compared with the sample without strontium (YC+Sr0), indicating that strontium benefits the proliferation of hMSCs. The YC+Sr0/Ca+Sr1 group also showed slight higher value than YC+Sr0 group but not statistically significant. Both YC+Sr0/Ca+Sr1 and YC+Sr1.0 groups presented comparable OD_450_ value to tissue culture plate group, indicating no significant cytotoxicity to hMSCs.

**
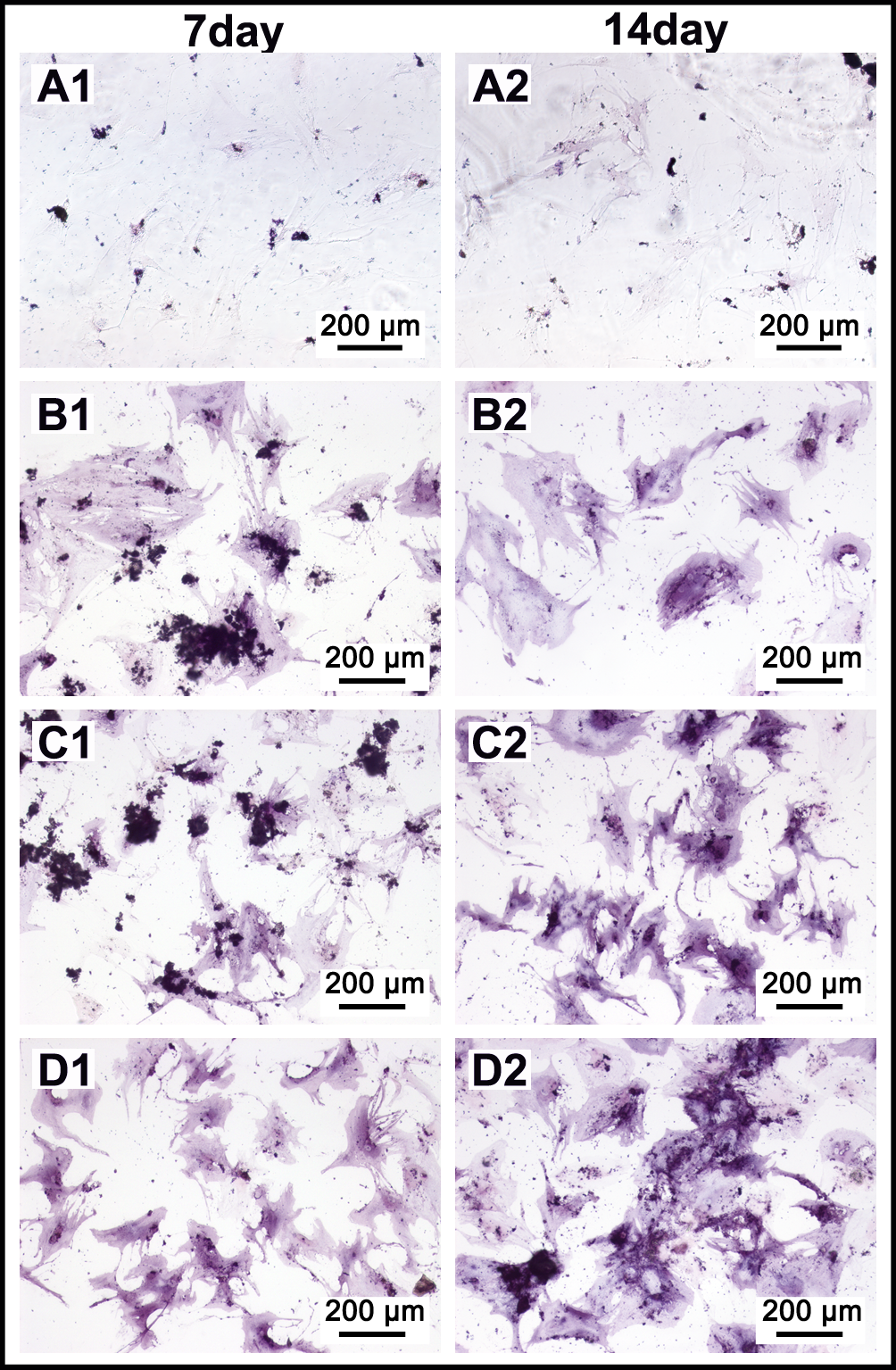
**

**Figure S4. ALP staining of the cultured hMSCs in (A) Control (without OGM induced CPMC), (B) YC+Sr0, (C) YC+Sr0/Ca+Sr1 and (D) YC+Sr1.0.**

ALP staining was used to examine the osteogenic differentiation status of hMSCs. More ALP-positive cells can be easily observed in Sr^2+^ containing groups, which indicates that strontium can remarkably enhance the osteogenic differentiation of the hMSCs. Moreover, there are more positive cells that contain intracellular purple granular dye observed in YC+Sr1.0 than the YC+Sr0/Ca+Sr1, indicating that the Sr^2+^ enriched in the core of YC+Sr1.0 played a better role in enhancing osteogenic differentiation process of hMSCs than the Sr^2+^ enriched in the shell of YC+Sr0/Ca+Sr1.
